# Epistatic interaction between the lipase-encoding genes *Pnpla2* and *Lipe* causes liposarcoma in mice

**DOI:** 10.1371/journal.pgen.1006716

**Published:** 2017-05-01

**Authors:** Jiang Wei Wu, Christoph Preuss, Shu Pei Wang, Hao Yang, Bo Ji, Gregory W. Carter, Rebecca Gladdy, Gregor Andelfinger, Grant A. Mitchell

**Affiliations:** 1 College of Animal Science and Technology, Northwest A&F University, Yangling, Shaanxi, China; 2 Division of Medical Genetics, Department of Pediatrics, CHU Sainte-Justine, Université de Montréal, Montréal, Québec, Canada; 3 The Jackson Laboratory, Bar Harbor, Maine, United States of America; 4 Cardiovascular Genetics, Department of Pediatrics, CHU Sainte-Justine, Université de Montréal, Montréal, Québec, Canada; 5 Division of General Surgery, the Lunenfeld-Tanenbaum Research Institute of Mount Sinai Hospital, University of Toronto, Toronto, Ontario, Canada; Centre for Cancer Biology, SA Pathology, AUSTRALIA

## Abstract

Liposarcoma is an often fatal cancer of fat cells. Mechanisms of liposarcoma development are incompletely understood. The cleavage of fatty acids from acylglycerols (lipolysis) has been implicated in cancer. We generated mice with adipose tissue deficiency of two major enzymes of lipolysis, adipose triglyceride lipase (ATGL) and hormone-sensitive lipase (HSL), encoded respectively by *Pnpla2* and *Lipe*. Adipocytes from double adipose knockout (DAKO) mice, deficient in both ATGL and HSL, showed near-complete deficiency of lipolysis. All DAKO mice developed liposarcoma between 11 and 14 months of age. No tumors occurred in single knockout or control mice. The transcriptome of DAKO adipose tissue showed marked differences from single knockout and normal controls as early as 3 months. *Gpnmb* and *G0s2* were among the most highly dysregulated genes in premalignant and malignant DAKO adipose tissue, suggesting a potential utility as early markers of the disease. Similar changes of *GPNMB* and *G0S2* expression were present in a human liposarcoma database. These results show that a previously-unknown, fully penetrant epistatic interaction between *Pnpla2* and *Lipe* can cause liposarcoma in mice. DAKO mice provide a promising model for studying early premalignant changes that lead to late-onset malignant disease.

## Author summary

Liposarcoma is an often fatal adult-onset tumor of fat tissue. Lipolysis, the central pathway of fat tissue metabolism, has been implicated in cancer. We generated mice that were deficient in two key enzymes of lipolysis, adipose triglyceride lipase (ATGL) and hormone-sensitive lipase (HSL). Strikingly, all mice with combined ATGL and HSL deficiency developed liposarcoma by 11–14 months of age. No liposarcoma occurred in single knockout or normal controls. Transcriptome analysis revealed that a subset of genes is dysregulated by 3 months of age. Our study reveals a novel epistatic interaction in fat cells between these two lipase genes and that causes a unique form of liposarcoma in mice. The double knockout mice provide a novel tool to study the early stages of liposarcoma development, prognostic markers and preventive treatments.

## Introduction

Liposarcomas are malignant tumors of fat. It is the commonest soft tissue sarcoma and the annual incidence is 2.5 cases per million [1]. Specific biomarkers for early diagnosis and specific curative treatments are not available for the disease. The WHO classification of liposarcomas identifies four categories: well-differentiated, myxoid, dedifferentiated (DDLS) and pleomorphic [2]. Well-differentiated liposarcoma is now considered to be a precursor to dedifferentiated liposarcoma [3, 4]. Distinct molecular changes are found in each category. For instance, myxoid liposarcoma has been associated with a specific t12;16 translocation and expression of a FUS-CHOP fusion protein [5]. Alternative lengthening of telomeres and other telomere maintenance mechanisms are active in pleomorphic, dedifferentiated and other liposarcomas [6–8]. Amplification of cyclin-dependent kinase 4 (CDK4) and murine double minute 2 (MDM2) occurs in well-differentiated and dedifferentiated liposarcomas [9], as does down-regulation of PTEN [10]. Some dedifferentiated liposarcomas show amplification of STAT6 [2]. Activation of the PI3K/AKT pathway has been implicated in liposarcomas [11–13] and loss of HIF-2a promotes liposarcoma growth [14]. Despite these major advances, important questions remain about the causes and the biology of liposarcomas.

Lipolysis, the pathway by which triglycerides are degraded, has been implicated in cancer, both as a source of fatty acids for tumor growth and as a mechanism of cancer-associated wasting [15–17]. Lipolysis has been studied biochemically in greatest detail in adipose tissue. Two major adipose lipases are adipose triglyceride lipase (ATGL) and hormone-sensitive lipase (HSL). ATGL, encoded by the *PNPLA2* gene on chromosome 11p15.5, is the main TG hydrolase of adipose tissue. It catalyzes the first step of lipolysis, i.e., the cleavage of triglyceride to diglyceride. Of note, deletion of *PNPLA2* is reported in well differentiated liposarcoma and sarcoma [18, 19]. HSL, encoded by *LIPE*, can catalyze the second step of lipolysis, the cleavage of diglycerides to monoglycerides. Deletions of the chromosome 19p13 region containing *LIPE* are frequent in DDLS and correlate with poor prognosis [18]. Although *PNPLA2* deletion has been reported in well differentiated liposarcoma and *LIPE* deletion, in DDLS with poor outcome [18], neither ATGL deficiency nor HSL deficiency reportedly causes liposarcoma in mice.

Increasing evidence suggests that cancer development may be driven by tissue-specific epistatic interactions due to mutations in multiple functionally-related genes [20, 21]. We generated three mouse lines with lipase deficiencies in adipose tissues, two single adipose knockout mice with deficiency of either ATGL or HSL plus double adipose knockout (DAKO) mice deficient in both ATGL and HSL. Strikingly, all DAKO mice developed a unique form of liposarcoma.

## Results

### Combined deletion of *Pnpla2* and *Lipe* in adipose tissue causes liposarcomas in mice

Complete deficiencies of ATGL and/or HSL in adipose tissue were confirmed in both white and brown adipose tissues by genomic Southern blotting (S1A Fig), Western blotting (S1B Fig) and real-time PCR of mRNA (S1C Fig). In isolated adipocytes, the rate of maximum beta adrenergic-stimulated lipolysis was markedly decreased in each single knockout line and was nearly unmeasurable in DAKO adipocytes (S2A Fig). Physiologically, DAKO mice were unable to maintain their blood glucose values with a normal postprandial fasting (S2B Fig), consistent with rapid depletion of carbohydrate reserves in the absence of lipid-derived energy from white adipose tissue (WAT). Cold tolerance, a measure of brown adipose tissue (BAT) function, was markedly reduced only in DAKO mice (S2C Fig).

**Fig 1:**
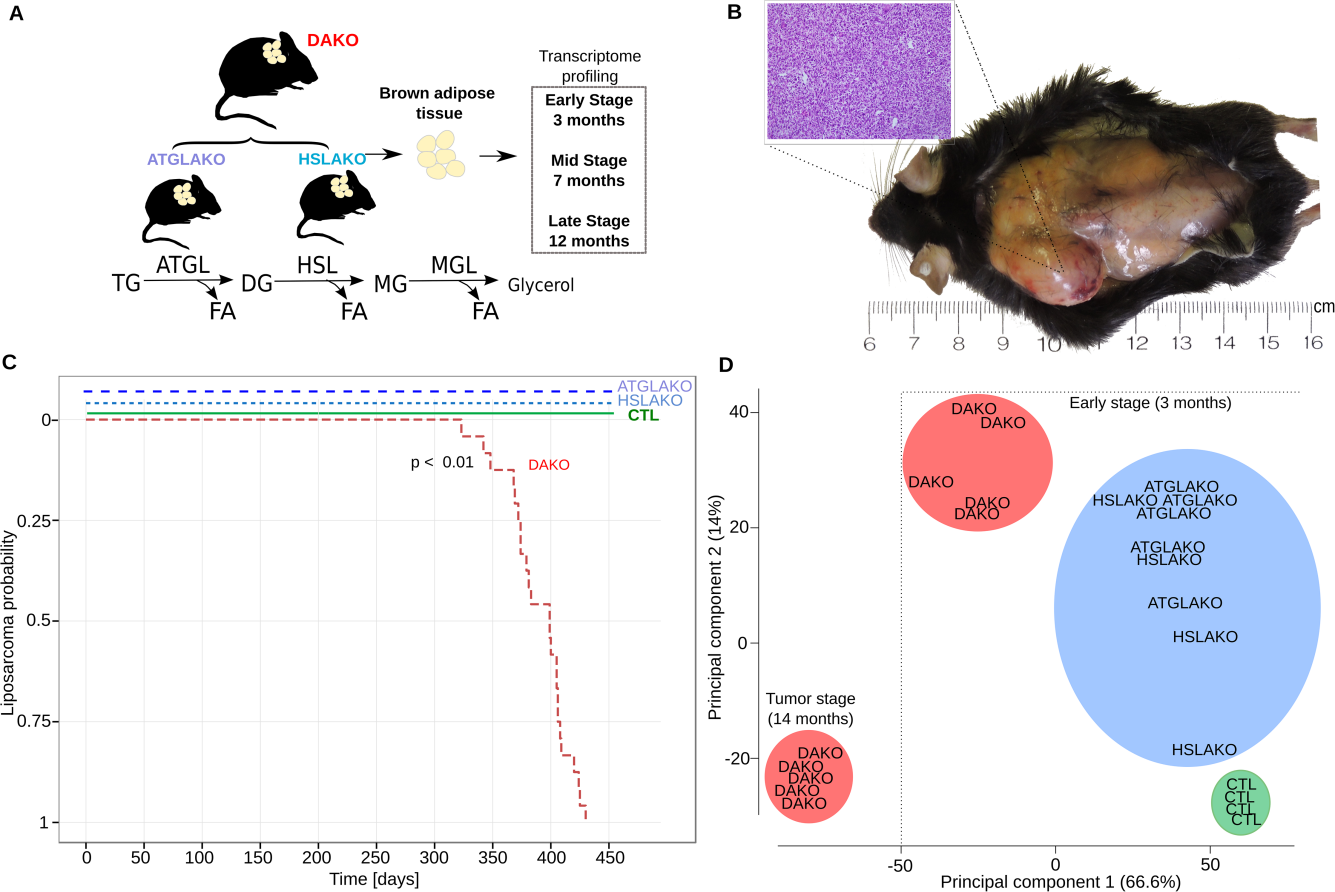
Characterization of lipase-deficient mice. (A) Experimental plan for generation of three different knockout mouse lines with lipolytic deficiency in adipose tissues. The positions of ATGL and HSL in the standard representation of the lipolytic pathway are shown below. (B) Liposarcoma in the left interscapular region of a 12-month-old DAKO mouse, gross and microscopic aspects. (C) Kaplan Meier plot displaying liposarcoma onset in the three knockout mouse lines and controls. (D) Principal component analysis (PCA) of whole transcriptome data, showing genotype-phenotype correlation for the early premalignant (3 months) and late (14 months) tumor stage. TG, triglycerides; DG, diglycerides; MG, monoglycerides; CTL, control.

ATGL and HSL catalyze the first two steps of lipolysis (Fig 1A). To test whether combined deficiency of ATGL plus HSL might promote liposarcoma (Fig 1A), 24 mice of each genotype were followed longitudinally. Both WAT and BAT were examined regularly by palpation and also directly at postmortem. No malignant tumors were found in WAT. In contrast, BAT of DAKO mice was hypertrophic and developed firm irregular tumors which invaded surrounding skin and tissue (Fig 1B). Histological evaluation of these tumors revealed liposarcoma (Fig 1B). Liposarcomas were detected between 11 and 14 months (24/24 mice, 100%) (Fig 1C). In contrast, neither single knockout line (ATGLAKO, HSLAKO) developed cancer (Fig 1C).

The clear genotype-phenotype correlation was confirmed with expression microarrays. Principle component analysis revealed that by 3 months, DAKO mice showed a clearly distinct transcriptional profile when compared to either of the single knockouts or to normal controls (Fig 1D). DAKO tumors were strikingly different from all other samples including those from pretumor DAKO mice.

### Premalignant DAKO BAT is morphologically abnormal and lacks UCP-1

**Fig 2:**
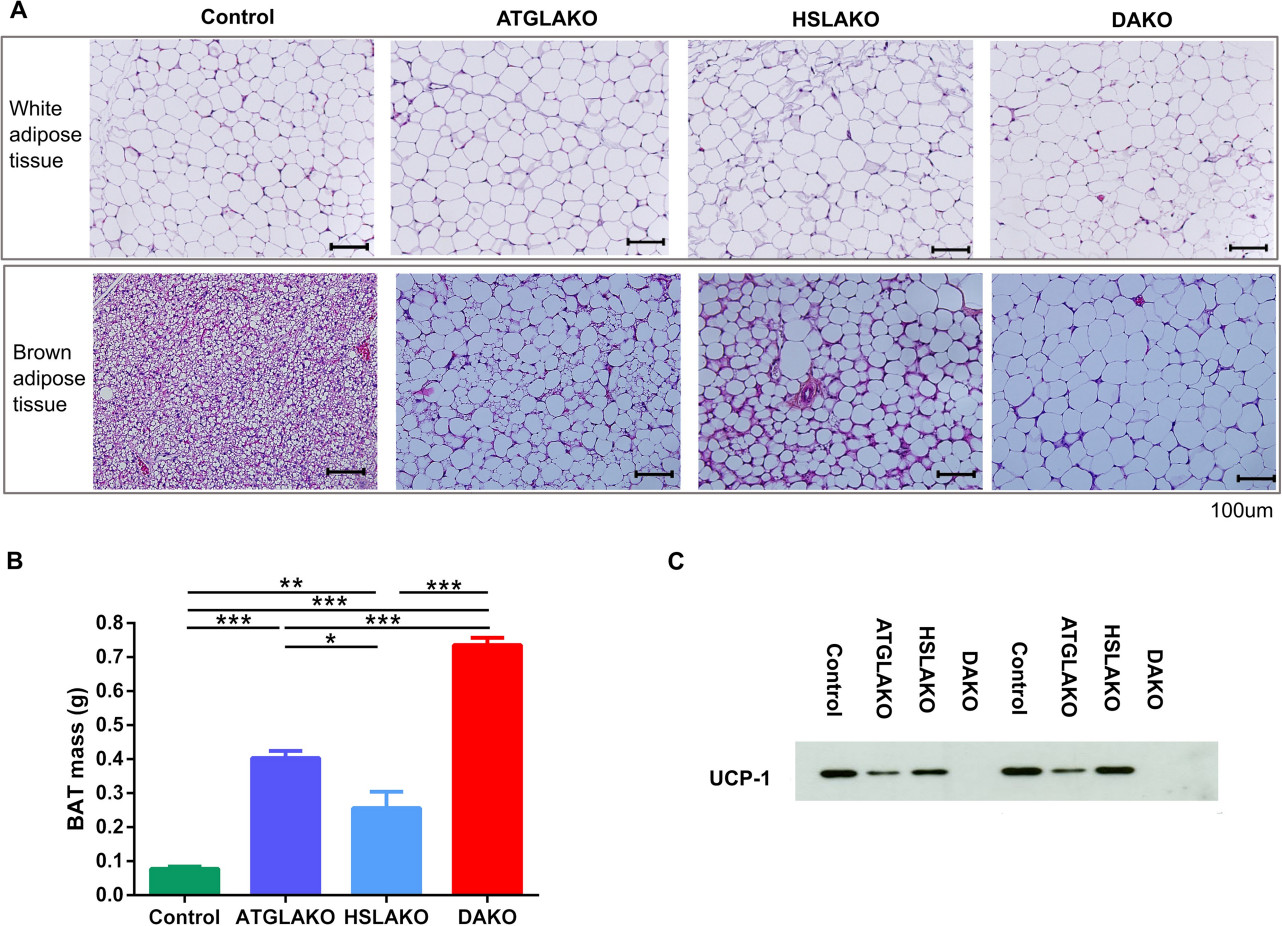
Tissue specific response related to genotype. (A) Histology of WAT and BAT, showing controls, single (ATGLAKO, HSLAKO) and double knockout (DAKO) mice. (B) Mass of interscapular brown adipose tissue in different genotypes (3 months). (C) Western blot of the brown adipose-specific marker, UCP-1 (3 months). *, p < 0.05; **, p < 0.01; ***, p < 0.001.

Morphologically, by 3 months, BAT of the single and double knockout strains clearly differed from that of normal controls (Fig 2A). In all three knockout lines, nearly all brown adipocytes contained a single large lipid droplet in contrast to the multilocular morphology of normal BAT. The interscapular BAT depots of each knockout line were hypertrophic, particularly in DAKO mice (Fig 2B). Also, uncoupling protein-1 (UCP-1), which normally is expressed strongly and specifically in BAT, was undetectable in BAT of DAKO mice, in contrast with normal controls and with each single knockout line (Fig 2C).

### Expression profiling of premalignant DAKO BAT

**Fig 3:**
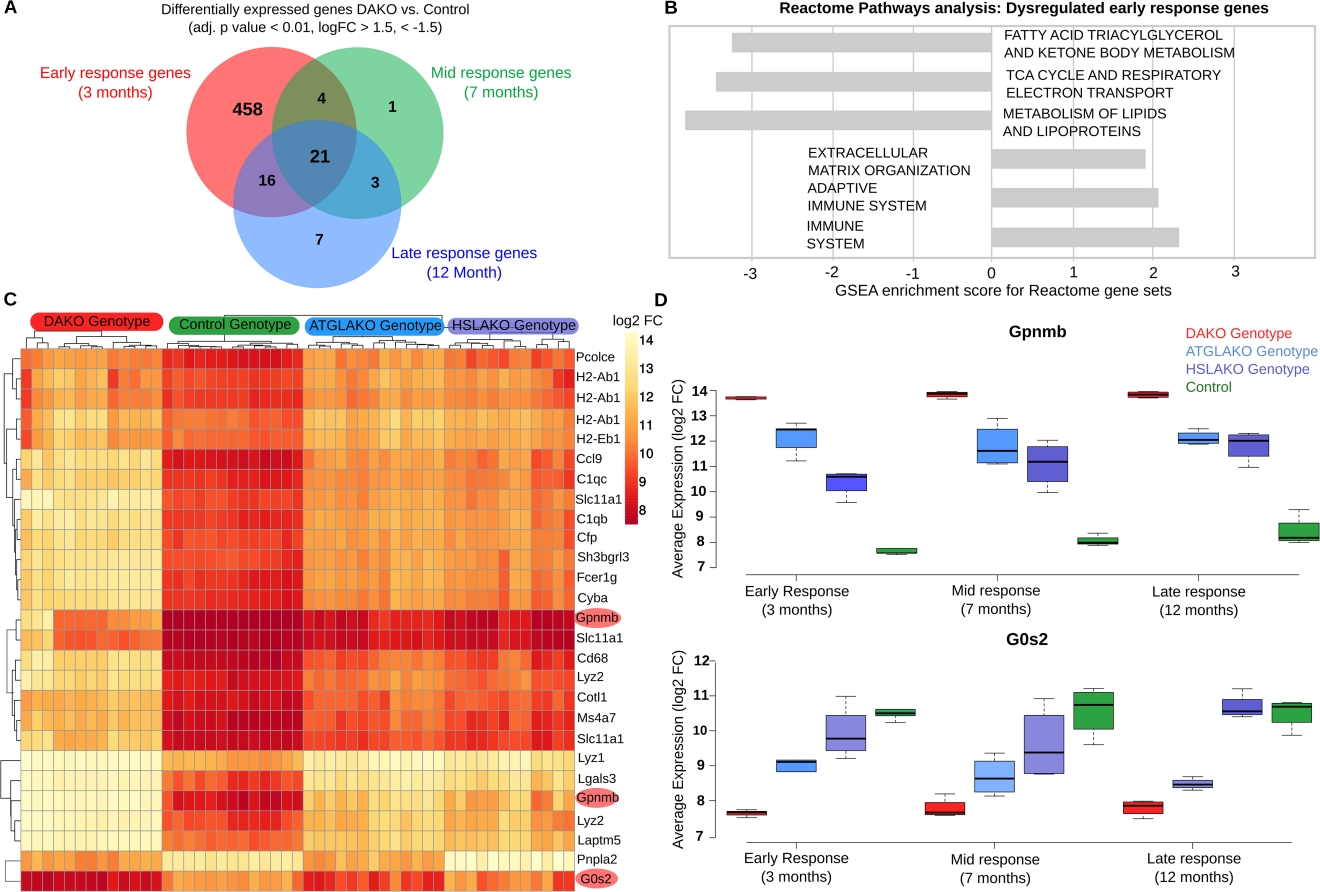
Early transcriptome analysis. (A) Venn diagram of intersections between differentially-expressed genes, comparing DAKO (n = 4) and WT mice (n = 4) at three different time points (3, 7 and 12 months). (B) Gene Set Enrichment Analysis for Reactome pathway, highlighting up- and down-regulated pathways for the differentially-expressed genes in BAT of 3-month-old mice. (C) Heatmap for all four genotypes, displaying the 21 genes that are consistently differentially-expressed between BAT of DAKO and control mice. Highlighted in red are Gpnmb and G0S2, the two most up- and down-regulated of these genes at all time points. (D) Boxplots indicating the expression levels of Gpnmb and G0s2 in the four mouse lines studied, at 3, 7 and 12 months.

In order to study the mechanism of tumor initiation and progression over time, whole-transcriptome profiling was performed for all genotypes illustrated in Fig 1A. Differential expression analysis between normal controls and DAKO mice at three time points (3, 7 and 12 months) revealed that 21 genes were consistently differentially expressed at each point (Fig 3A). The greatest difference occurred at 3 months, when 499 genes were found to be differentially expressed between normal control and DAKO BAT. Gene set enrichment analysis (GSEA) using reactome pathways [22, 23] identified six pathways which were significantly enriched at an adjusted p value cutoff of p < 0.05 (Fig 3B). Down-regulation occurred for the gene sets of fatty acid, triacylglycerol and ketone body metabolism, the tricarboxylic acid (TCA) cycle and respiratory chain and genes of lipid metabolism. In contrast, genes involved in the immune response, particularly adaptive immunity, were up-regulated. Together, these results in premalignant DAKO BAT suggest a global down-regulation of oxidative energy metabolism and up-regulation of the immune response.

Heat-map clustering of the 21 genes that are consistently differentially-expressed in DAKO and control BAT reveals a clear genotype-phenotype correlation, with marked differences between DAKO and normal control BAT and intermediate profiles in each single knockout line (Fig 3C). Among these genes, the two with the greatest up- and down-regulation in liposarcoma were glycoprotein nonmetastatic melanoma protein B (*Gpnmb*) and G0–G1 switch gene 2 (*G0S2*), respectively.

Gpnmb, a circulating glycoprotein, has been identified as a therapeutic target for several cancers [24]. Therapeutic suppression of Gpnmb is currently in phase 2 clinical trials for several types of cancer (ClinicalTrials.gov Identifier: NCT01997333, NCT01156753, NCT02713828, NCT02487979, NCT02302339, NCT02280785, NCT00921570, NCT02363283). Gpnmb is the top up-regulated gene in malignant and also premalignant DAKO BAT, compared to each of the other three genotypes (Fig 3C and 3D). Of note, plasma Gpnmb levels were also significantly (~3 fold) elevated in malignant and also premalignant DAKO mice compared to single knockout and normal controls, as early as 3 months of age, long before detectable liposarcoma development (S3 Fig).

Because Gpnmb is expressed in macrophages [25, 26], we explored whether macrophage expression of Gpnmb might account for its high level in DAKO BAT. Macrophage-related transcripts were found to be higher in HSLAKO BAT than in BAT of DAKO or in the other control lines (S4A Fig), but DAKO plasma shows higher levels of Gpnmb and DAKO BAT shows higher levels of *Gpnmb* expression than do corresponding samples from HSLAKO mice or from the other genotypes (S3 Fig). Therefore *Gpnmb* expression is high in DAKO BAT, and more so than anticipated from the observed level of macrophage infiltration. To explore whether adipocytes express Gpnmb, we performed in vitro differentiation of cultured brown adipocytes isolated from intrascapular DAKO BAT and also of cultured NIH 3T3-L1 white preadipocytes. Gpnmb was detectable after differentiation in both DAKO brown adipocytes and in 3T3-L1 cells (S4B Fig). Taking these observations together, it is likely that the high levels of Gpnmb in DAKO mice arise directly from adipocytes, although a fraction may derive from macrophages.

*G0s2*, the endogenous inhibitor of ATGL [27], is epigenetically silenced in several types of cancer [28, 29]. Interestingly, G0s2 was the transcript showing the greatest down-regulation in DAKO mice compared with normal controls and single knockout mice (Fig 3C and 3D).

Also, well-known liposarcoma markers such as MDM2 and p16/cdkn2 were detected. MDM2 is highly expressed in DAKO BAT at all ages studied (S5 Fig). The expression of two inhibitors of Cdk4, Cdkn2a and Cdkn2b, was significantly increased, as a late event, present only in DAKO liposarcomas (S5 Fig). Other cancer-associated genes such as *Top2a*, *Ptn*, *Ptk7*, *Tnc* and *Mmp14* were highly expressed in DAKO mice (S5 Fig). Therefore, the expression of some known liposarcoma-related genes coincides with DAKO liposarcoma development.

### High expression of angiogenesis-related genes in DAKO tumors

To further explore the late onset of tumor formation, we compared premalignant and malignant DAKO BAT. Morphologically, after 7 months, extensive necrosis is observed in BAT of DAKO mice (S6A Fig). Compared to late stage non-tumor BAT samples of DAKO mice, Gene Set Enrichment Analysis for Reactome pathway in DAKO liposarcomas revealed that the pathway with the greatest relative up-regulation of gene expression in DAKO liposarcomas was angiogenesis (S6B Fig), In this pathway, the highest up-regulation was seen for *Esm1*, encoding Endocan, expression of which has been reported to be increased up to 30-fold in human liposarcoma (SW872) cell lines [30, 31]. Other genes of angiogenesis that show upregulation in DAKO liposarcoma are *Tnc*, *Ptn* and *Tnfrsf12a* (S6B Fig). Together, these results suggest that angiogenesis is a key feature of DAKO liposarcoma development.

### Differences in gene expression between WAT and BAT were reduced in DAKO mice

Morphologically, BAT samples from single knockout and DAKO mice show monolocular lipid droplets, as in WAT (Fig 2A). We compared expression data from WAT and BAT of three-month-old mice (S7 Fig). The number of genes that are differentially-expressed between brown and white adipose tissues is smaller in DAKO mice (114) than in normal controls (435), ATGLAKO (228) or HSLAKO mice (350) (S7A Fig). A subset of 48 genes is differentially expressed between BAT and WAT in all genotypes studied (S7B Fig). Based on these markers we performed time-course analyses. A subset of these BAT markers (Ucp1, Cidea, Cox8b) decreased progressively in DAKO BAT (S8 Fig). Of note, Pparγ, a critical regulator of identity and differentiation in white and brown adipocytes [32], is slightly but significantly down-regulated in 7- and 12-month-old DAKO BAT (S8 Fig).

### Gene expression and copy number changes in DAKO liposarcoma and in human cancers

**Fig 4:**
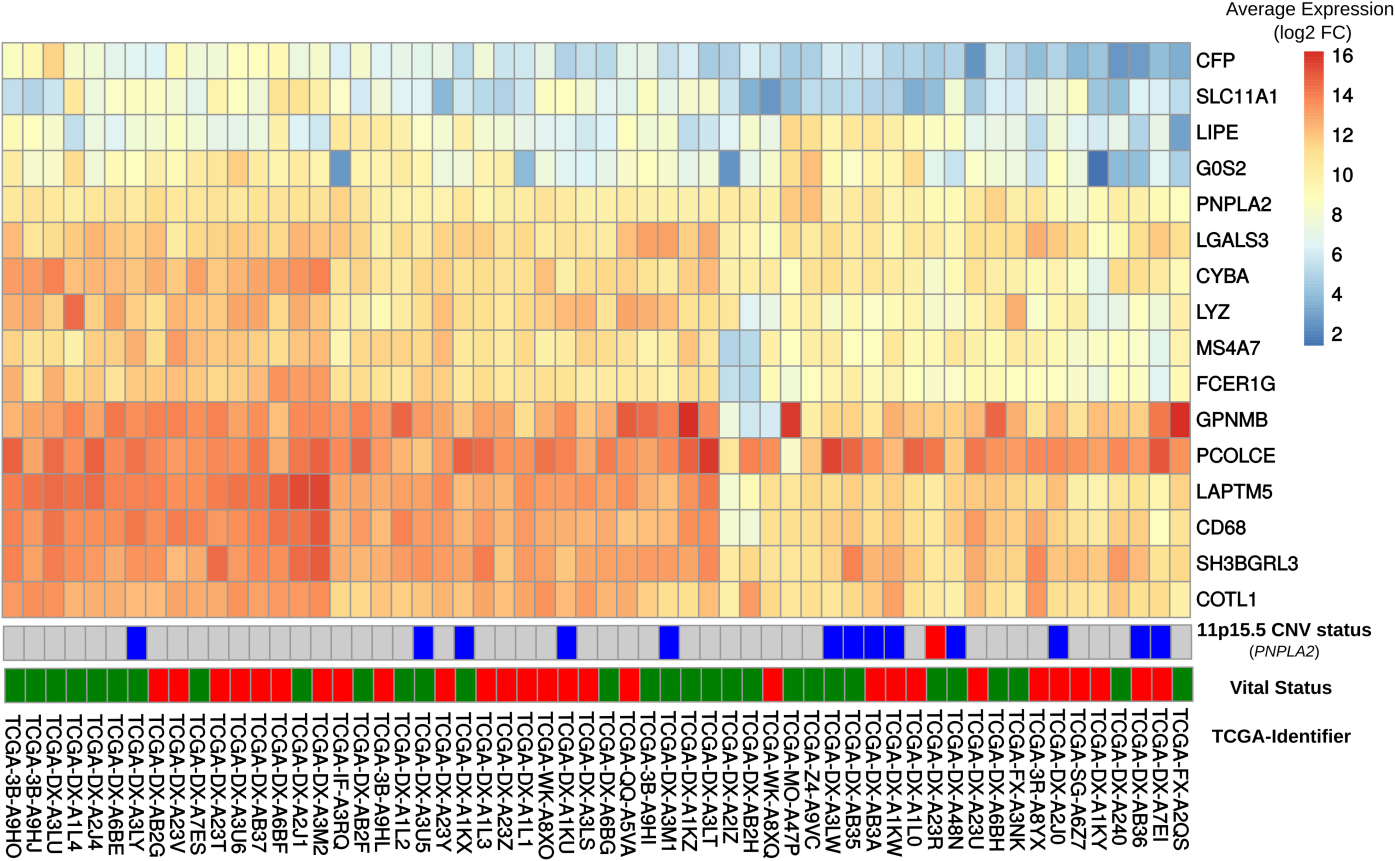
Comparison of gene expression in DAKO adipose tissue and in human dedifferentiated liposarcomas. Heatmap displaying gene expression patterns for all human-mouse orthologous genes in the publicly-available data set of 58 dedifferentiated liposarcomas. The vital status line indicates dead (red) or alive (green).

In order to explore whether some of the changes observed in DAKO liposarcoma might also be present in some human liposarcomas and other tumors, gene expression data from 58 DDLS patients from the TCGA data [33] were analyzed. Analysis of the orthologues of the 21 genes that were consistently differentially-expressed in DAKO BAT, revealed that *LIPE*, *PNPLA2* and *G0S2* are also among the five most down-regulated genes in human liposarcoma (Figs 3C and 4). Therefore, tissue specific inactivation of *Pnpla2* and *Lipe* causes liposarcoma in mice, and down-regulation of *PNPLA2* and *LIPE* expression occurs in human DDLS.

We also examined the expressions of *GPNMB* and *PCOLCE* in the 58 DDLS samples. No significant difference was found in the small number of samples, although there was high variability among the samples. Interestingly, among the 497 different types of cancer-related expression profiles in TCGA, *GPNMB* and *PCOLCE* were highly expressed in the two available pleomorphic liposarcoma samples (S9A Fig) and in sarcoma samples in general (S9B Fig).

In addition, copy number analysis for 265 patients with soft tissue sarcoma from the TCGA dataset was performed to test the hypothesis that *PNPLA2* and *LIPE* might be tumor suppressors in some tissues. The loss of chromosome 11p15.5 harboring *PNPLA2* was significantly associated (p < 0.01, chi-square) with soft tissue sarcomas (S10 Fig). In contrast, copy number changes of the region on chromosome 19q13 containing *LIPE* were not observed (S10 Fig).

## Discussion

Our study showed that the combined deficiency of the two major lipases of adipose tissue, ATGL and HSL, causes a unique form of liposarcoma with complete penetrance in mice. This clearly reveals a positive synergistic epistatic interaction between the *Pnpla2* and *Lipe* genes, because neither single lipase deficiency causes liposarcoma.

There is growing interest in epistasis in cancer. Most cancers cannot be solely explained by single, highly-penetrant genetic changes [20, 21]. Large scale genetic analysis of human cancers has revealed co-enrichment (~5 to 16 fold) of genes whose products are known to physically or functionally interact in a tissue specific manner [20]. However, to date, few examples of functionally-proven biochemical interactions between members of the same metabolic pathway have been directly linked to epistatic mechanisms of cancer [21].

Each lipase of the lipolytic pathway shown in Fig 1 has clearly been related to cancer. Activation of monoacylglycerol lipase (MGL), which catalyzes the last step of lipolysis, promotes the progression of several cancers [34]. MGL has multiple physiological roles in addition to the cleavage of monoacylglycerols [35]; the mechanism by which MGL activation promotes cancer is unclear. ATGL is now linked to cancer in many ways. Deficiency of CGI-58, the coactivator of ATGL encoded by *ABHD5*, results in increased tumorigenesis and malignant transformation in mice and *ABHD5* deletions occur in several human cancers [36]. G0S2, the endogenous inhibitor of ATGL, is epigenetically methylated in several cancers [28], has been considered to function as a tumor suppressor [37] and is markedly down-regulated in DAKO adipose tissue and DAKO liposarcoma. Al-Zougbi et al [19] recently reported lung cancer in 25% of ATGL-deficient mice that had prolonged survival. This group also studied ATGL copy number in a variety of human malignancies, and tested ATGL expression histochemically in human lung adenocarcinoma and squamous cell carcinoma, pancreatic intraductal neoplasia and adenocarcinoma and uterine leiomyosarcoma. They found lower ATGL expression in these cancers than in the corresponding normal tissues. The authors hypothesized that reduction of ATGL might reduce fatty acid availability and oxidation by the tumor cells, increasing their dependence on other energy sources like glucose and glutamine. A role for HSL in cancer development is less well documented than for MGL and ATGL, but a study of patients with well-differentiated and dedifferentiated liposarcoma showed that loss of the chromosome 19q13 region containing *LIPE* occurred frequently in DDLS, and that this was associated with decreased survival [18]. HSL deficiency is clearly essential for the development of DAKO liposarcomas. Taken together, these observations suggest that disruption of lipolytic pathway enzymes may occur widely in cancer and sometimes can be the primary and sufficient cause of cancer, as in DAKO liposarcoma.

ATGL and HSL are closely related metabolically. Functional overlap between these two lipases, as directly shown by lipolysis in isolated DAKO adipocytes and by intolerance to short fasting in DAKO mice, provides a potential mechanism for the epistatic phenomenon that occurs when both are deficient. During revision of this article we noted the description by Bi *et al* [38] of a mouse model of liposarcoma caused by adipocyte-specific overexpression of Notch1 intracellular protein (N1ICP). The authors conclude that deficiency of fatty acids within adipocytes and reduction of PPAR-γ signaling are critical events in the development of that form of liposarcoma. Intriguingly, although N1ICP-related liposarcomas occur in white adipocytes whereas DAKO liposarcoma occurs in brown adipose tissue, both the DAKO and N1ICP-related liposarcomas occur with complete penetrance, and in both, intracellular fatty acid pools and PPAR-γ signaling are predicted to be reduced. The discovery of the precise mechanism(s) by which combined deficiencies of ATGL and HSL cause liposarcoma will require further study. In DAKO BAT, major changes in gene expression are present by 3 months. This premalignant state may provide clues to the conditions under which DAKO liposarcomas develop.

In conclusion, our findings suggest that a synergistic epistatic interaction between two functionally related genes of the lipolysis pathway plays an important role in cancer development. The DAT mice described here provide a novel system for deciphering the early stages of development of liposarcoma and for testing prognostic markers and preventive treatment strategies.

## Materials and methods

### Ethics statement

Experiments were approved by Animal Facility Committee of CHU Sainte-Justine Hospital (protocol 620) according to the guidelines of the Canadian Council on Animal Care (http://www.ccac.ca/en_/).

### Creation of ATGLAKO, HSLAKO and DAKO mice

Four mouse strains were used: normal controls, mice deficient in adipose tissue for ATGL (ATGLAKO), HSL (HSLAKO) or both (DAKO). The creation of ATGLAKO mice was described previously [39]. HSLAKO mice were homozygous for a floxed allele of *Lipe* and harbored a transgene, from which Cre is expressed from a *Fabp4* promoter, which is active in adipose tissues. Further details about the creation of HSLAKO mice are provided in S1 Text. DAKO mice were created by breeding of the two single knockout lines. Mice expressing only the Fabp4-Cre transgene were used as controls. Each targeted allele had been transferred for at least 8 generations to a C57BL/6J background.

### Mouse breeding and handling

Mice were raised with a light-dark cycle with lights on at 7am and off at 7pm, and free access to water and food (no. 2019, Teklad Global Rodent, 9% fat; Harlan Laboratories, Madison, WI). Mice were housed at 21° C unless otherwise specified. For sacrifice, mice were anesthetized with sodium pentobarbital (Somnotol; MTC Pharmaceuticals, Hamilton, Ontario, Canada). After cardiac puncture, organs were rapidly removed, weighed, and frozen. Experiments were approved by the Canadian Council on Animal Care-accredited animal facility committee of CHU Sainte-Justine Hospital.

### Western blotting & plasma GPNMB measurement

Western blotting was performed as described [40]. The following antibodies were used: ATGL (#10006409–1, Cayman Chemical), HSL [40], UCP-1 (# RB-10599-P0, Lab vision corporation) and GPNMB (#AF2330-SP, R&D Systems). Plasma levels of GPNMB soluble protein were measured using the commercially available ELISA kit from ORIGENE (EA100800).

### Lipolysis in isolated adipocytes

Isolation of adipocytes and measurement of lipolysis were performed as described [39].

### Fasting tests in lipase-deficient mice

Six mice of each genotype, aged 3 months, were fasted starting at 7am. Plasma glucose was measured at 12 noon.

### Thermogenesis

Six mice of each genotype, aged 2.5 months, were placed at 4°C. Five hours after food removal, body temperature was measured using a rectal thermometer (BAT-10 Thermometer; Physitemp Instruments, Inc., Clifton, NJ).

### Whole-transcriptome analysis

Interscapular BAT from 3, 7 and 12-month old mice for tumor and non-tumor BAT and perigonadal WAT was rapidly obtained from anesthetized 5h fasted mice, then was snap frozen in liquid nitrogen. RNA was extracted using the RNeasy Lipid Tissue Mini Kit (QIAGEN, Cat. No.74804). Illumina mouse Ref-8 v2.0 Expression BeadChips were used for whole-transcriptome profiling. http://support.illumina.com/array/array_kits/mouseref-8_v2_expression_beadchip_kit/questions.html. Raw expression data was obtained as output from the BeadArrray system via the Beadstudio software interface. Quality control and preprocessing of the raw data was performed using the R & Bioconductor software suite. Linear models for microarray data (limma) were used to identify significant expression differences of genes between biological replicates. Probe identifiers were mapped to HGNC symbols and Refseq identifiers for subsequent pathway and Gene Set Enrichment Analysis. For differential gene expression analysis, all possible comparisons between the three genotypes and the normal controls were designed. Genes designated as having significant differential expression had a p-value threshold of 0.01 after correction for multiple testing by the false discovery rate method (FDR) [41]. Genes were sorted according to the empirical Bayes log odds of differential expression (B value) and the adjusted p values. Hierarchical clustering of genes based on Pearson’s correlation coefficients revealed groups of genes with similar expression patterns across genotypes. Groups of differentially-expressed genes were displayed as heatmaps or Venn diagrams. Gene Set Enrichment analysis was performed using the standard parameters for the Reactome pathway hallmark data set (www.broadinstitute.org/gsea/msigdb/collections) [22, 23].

### Expression and CNV data from sarcoma patients

Gene expression and CNV data from 265 soft sarcoma patients (SARC cohort) and 972 breast cancer patients was obtained from the TCGA dataset [33] via the firebrowse data repository (www.firebrowse.org). Expression data was analyzed for a subset of 58 patients with dedifferentiated liposarcoma based on human-mouse ortholog comparisons in the DAKO mouse model. CNV data was processed using GISTIC2 [41] and estimated values were assigned to the following thresholds of -2, -1, 0, 1, 2, representing homozygous deletion, single copy deletion, diploid normal copy, low-level copy number amplification, or high-level copy number amplification, respectively. The gene visible data source (www.genevisible.com), containing expression profiles of 497 cancers, and the TCGA dataset were used to visualize cancer-specific expression profiles for PCOLCE and GPNMB. CNV data from 265 soft tissue sarcoma patients from the TCGA resource was analyzed to identify gene deletions and losses associated with chromosomal regions harboring PNPLA2 and LIPE.

### Culture and differentiation of interscapular brown adipocytes and NIH 3T3-L1 cells

Interscapular brown adipocytes from 3-day-old mice were isolated and cultured, then differentiation was induced as described [42]. NIH3T3-L1 cells were cultured and differentiation was induced as described [43].

### Web resources

Reactome pathway hallmark data set; www.broadinstitute.org/gsea/msigdb

Broad portal for TCGA cancer data set; www.firebrowse.org

Genevisible expression data set; www.genevisible.com

## Supporting information

S1 Text Materials and methods for creation of mice with tissue-specific HSL deficiency. (DOCX)

S1 Fig Knockout of *PNPLA2* and *LIPE* in adipose tissue. Efficient knockout of *Pnpla2* and *Lipe* in adipose tissues. (A) Southern blot demonstrating Lipe gene deletion in HSLAKO mice. (B) Western blot showing levels of ATGL and HSL in perigonadal WAT and interscapular BAT according to genotype. (C) mRNA expression of Pnpla2 and Lipe in brown adipose tissue (BAT) and white adipose tissue (WAT), measured by qPCR.
(TIF)

S2 Fig Functional consequences of ATGL and HSL deficiencies in WAT and BAT in single and double knockout mice. (A) Lipolysis in isolated adipocytes. Adipocytes were isolated from the perigonadal fat pad and lipolysis was maximally stimulated by incubation with the beta-3 adrenergic agonist, CL316, 243. (B) Maintenance of plasma glucose level during fasting. 3-month-old mice of the indicated genotypes were fasted for 5 hours. (C) Cold tolerance, measured as body temperature after housing at 4°C for 5 hours. *, p < 0.05; **, p < 0.01; ***, p < 0.001.
(TIF)

S3 Fig Circulating levels of Gpnmb. Gpnmb levels of 3- and 12-month-old mice of the indicated genotypes. *, p < 0.05; **, p < 0.01; ***, p < 0.001.
(TIF)

S4 Fig The origin of Gpnmb expression in adipose tissue samples. (A) Expression of macrophage marker mRNAs in brown adipose tissue of mice. (B) Western blot showing Gpnmb expression before and after in vitro differentiation, in cultured DAKO brown adipocytes from DAKO mice and in NIH-3T3 L1 cells. *, p < 0.05; **, p < 0.01; ***, p < 0.001.
(TIF)

S5 Fig Heatmap of the expression of genes related to liposarcoma and other cancers. Heatmap showing the sarcomas and other cancer-associated gene expression in four genotypes of mice studied. Sample type (liposarcoma, BAT) and genotype are indicated in the dendrogram on the top. The genes in question are listed on the right.
(TIF)

S6 Fig Transcriptomic profiling of tumor tissue. (A) Haematoxylin-eosin stained DAKO BAT at four time points (3, 7, 9 and 12 months). (B) Differential expression analysis, comparing pre-cancerous BAT and liposarcoma tissue, highlighting the most up- and down-regulated genes identified in transcriptome analysis. Candidate genes involved in angiogenesis are displayed as boxplots for four different time points: early (3 months), mid (7 months), late (12 months) and tumor (11–14 months).
(TIF)

S7 Fig Transcriptomic profiling of brown and white adipose tissue. (A) Venn diagram highlighting differentially expressed genes between brown and white adipose tissues for all possible genotype combinations in three-month-old mice. (B) Heatmap displaying the subset of 48 differentially-expressed genes for all genotypes. Commonly-used markers for BAT (Ucp1, Cidea, Cox8b) are highlighted in red.
(TIF)

S8 Fig Expression changes in premalignant brown adipose tissue. Time course analysis of BAT markers Ucp1, Cidea, Pparγ and Cox8b prior to tumor development in the course of 12 months. Significant down regulation of genes was assessed by comparing expression means using ANOVA. **, p < 0.01; *, p < 0.05.
(TIF)

S9 Fig GPNMB and PCOLCE as prognostic markers for liposarcoma. (A) Data from the genevisible dataset containing 497 different cancer expression profiles highlighting the 10 cancers with the highest expression of the two potential markers GPNMB and PCOLCE. (B) Data from the TCGA dataset, visualized by the Firebrowse website available through the Broad Institute indicating expression levels of GPNMB and PCOLCE across all available cancer subtypes. SARC, sarcoma
(TIF)

S10 Fig Copy number variation in 265 human sarcomas. (A) Cosmic annotation of CNVs in 265 patients with soft tissue sarcomas. The 11p15.5 region containing PNPLA2 is boxed. (B) Chromosomal region 11p harboring PNPLA2 has a significantly increased deletion frequency. Chromosomal region 19q, containing LIPE, does not show copy number variation.
(TIF)
