# Supplementary material for: Epistatic interaction between the lipase-encoding genes Pnpla2 and Lipe causes liposarcoma in mice
Source: PLoS Genet. 2017 May 1;13(5):e1006716. doi: 10.1371/journal.pgen.1006716 (PMC5432192; doi:10.1371/journal.pgen.1006716)
Supplement: S1 Text — (DOCX) [file pgen.1006716.s001.docx]

**Supporting information**

**Materials and Methods**

**Creation of mice with tissue-specific HSL deficiency**

**Targeting Vector Construction**

The HSL targeting vector contains LoxP sites flanking a bacterial neomycin resistance gene (Neo) and exon 1 of *Lipe*. *Lipe* sequences were from the 7.5 kb BamHI subclone of the previously-described 129 mouse *Lipe* genomic clone (PMID: 9060404). For the 5’ lox site and Neo cassette, we modified the pMC1neo PolyA vector (Stratagene, La Jolla, CA). First, the unique XhoI site, situated 3’ to the Neo cassette, was replaced by an AflII site. Then, the BamHI and HindIII sites of the pMC1neo PolyA polylinker, 5’ to the Neo cassette, were digested and blunted. A synthetic oligonucleotide, containing a 5’ AflII site and the LoxP sequence, was cloned into the blunted BamHI and HindIII site. The AflII fragment from the resulting vector contained a 5’ Lox sequence plus the Neo cassette. This fragment was cloned into the naturally-occurring AflII site of the *Lipe* genomic subclone, 763 bp 5’ of the initiation ATG codon in *Lipe* exon 1. For the 3’ LoxP sequence, a synthetic oligonucleotide containing the LoxP sequence flanked by EcoRV sites was introduced into the naturally-occurring EcoRV site of the *Lipe* genomic subclone, 1174 bp downstream of the initiation ATG codon of *Lipe*. The final ~8.5 kb targeting insert containing the Neo cassette and the floxed *Lipe* exon 1, was cloned downstream from a thymidine kinase cDNA with a phosphoglycerate kinase promoter. The *Lipe* fragment has a 0.9 kb short arm and a 4.8 kb long arm.

**HSL (*Lipe*) gene targeting in embryonic stem (ES) cells**

Targeting of *Lipe* was performed in J1 ES cell line from 129S/VJ mouse, using linearized targeting vector, as described (PMID: 17070019).

**Genotyping of the targeted *Lipe* allele by PCR and Southern Blotting**

ES cell colonies detected by positive-negative selection were first screened by PCR. The ES cells were washed with PBS, and then lysed. The cell lysates were digested with proteinase K as described (PMID: 9817922). 20% of the lysate was used for PCR amplification. PCR conditions were as described previously (PMID: 9817922) using the sense primer LipM100 (5’-AGTCTGAGCAAGGAGAGCCT -3’), which is outside the targeting vector, corresponding to residues -1759 to -1739 nt upstream of ATG and the antisense primer Neo-4 (5’-GATTCGCAGCGCATCGCCTT). The presence of a 1.2-kb fragment was diagnostic for the targeted allele. We found 27 targeted clones by screening 60 ES cell colonies (45%). The targeted ES cell clones were confirmed by Southern blotting. ES cell genomic DNA (5 µg) was digested with EcoRI and probed with a 1.6 kb BamHI fragment (PMID: 9060404), located outside the targeting vector and corresponding to residues -3355 to -1796 nt upstream of ATG. The probe detected 9.1-kb and 7.7-kb fragments for the normal and targeted alleles, respectively.

**Production of mice with HSL deficiency in adipose tissue**

Targeted ES cell clones were microinjected into C57BL/J blastocysts and transferred to pseudopregnant recipients. Chimeras were bred to C57BL/J mice. Agouti-colored offspring were genotyped to identify heterozygotes (+/flox). In order to obtain excision of exon 1 of the *Lipe* gene in adipose tissue, a Cre-recombinase transgene driven by the Fabp4-Cre promoter (B6.Cg-Tg (Fabp4-Cre) 1 Rev/J, 005069, The Jackson Laboratory, Bar Harbour, ME) was introduced by breeding with HSL^+/flox^ mice. Mice heterozygous for the targeted *Lipe* allele and for the Cre transgene (HSL^+/flox^ Cre^+^) were interbred to obtain homozygotes for the floxed *Lipe* allele that expressed the Cre transgene (HSL^flox/flox^ Fabp4-Cre ^+^). HSL^flox/flox^ Fabp4-Cre ^+^ mice were designated as HSLAKO (HSL adipose knockout) mice. HSL^+/+^ Fabp4-Cre ^+^ mice served as controls.

**PCR detection of Cre transgenes**

This was performed with Cre-1 (5’-GATGGACATGTTCAGGGATC-3’ and Cre-2 (5’-AGCTTGCATGATCTCCGGTA-3’) primers. Cre-1 is a sense primer, corresponding to nucleotides 80 to 100 bp of the Cre cDNA; and Cre-2 is an antisense primer, corresponding to residues 916 to 936 bp.
