# Supplementary figures and images for: Epistatic interaction between the lipase-encoding genes Pnpla2 and Lipe causes liposarcoma in mice
Source: PLoS Genet. 2017 May 1;13(5):e1006716. doi: 10.1371/journal.pgen.1006716 (PMC5432192; doi:10.1371/journal.pgen.1006716)

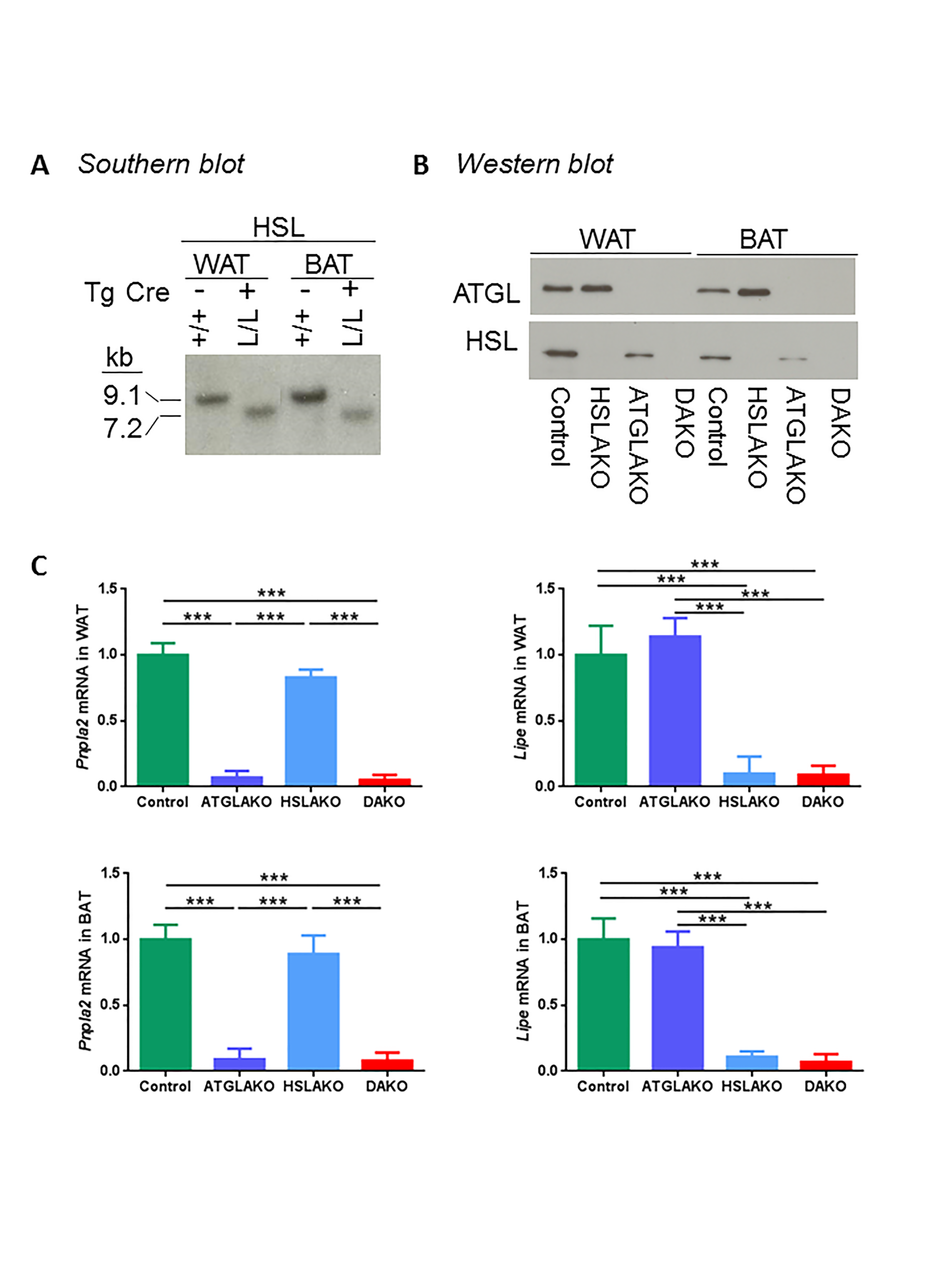

Supplement: S1 Fig — Efficient knockout of Pnpla2 and Lipe in adipose tissues. (A) Southern blot demonstrating Lipe gene deletion in HSLAKO mice. (B) Western blot showing levels of ATGL and HSL in perigonadal WAT and interscapular BAT according to genotype. (C) mRNA expression of Pnpla2 and Lipe in brown adipose tissue (BAT) and white adipose tissue (WAT), measured by qPCR. (TIF) [file pgen.1006716.s002.tif]

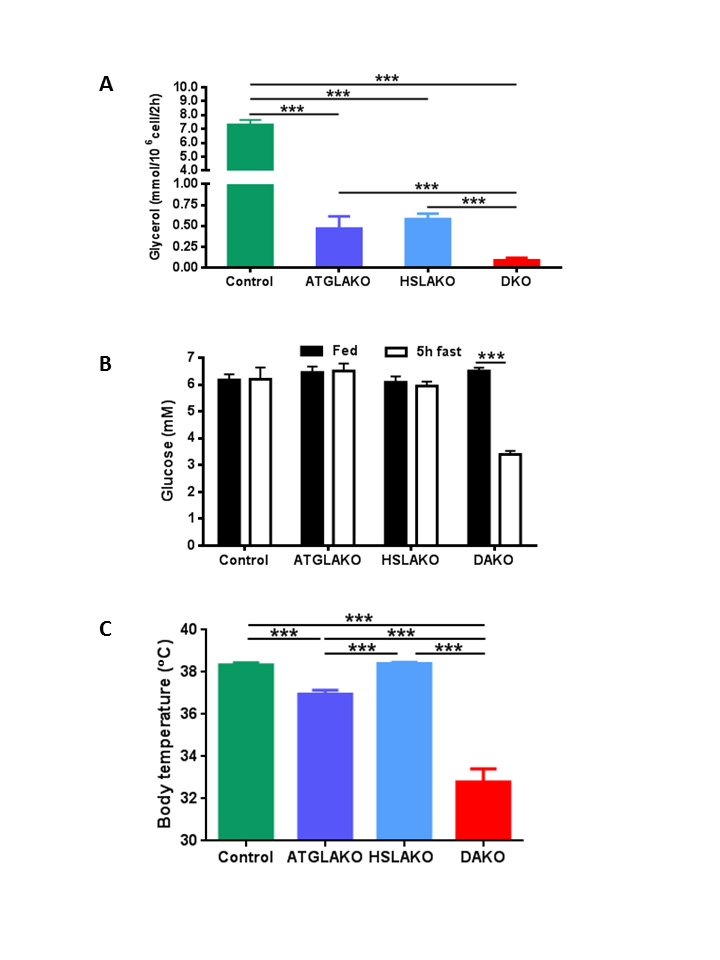

Supplement: S2 Fig — (A) Lipolysis in isolated adipocytes. Adipocytes were isolated from the perigonadal fat pad and lipolysis was maximally stimulated by incubation with the beta-3 adrenergic agonist, CL316, 243. (B) Maintenance of plasma glucose level during fasting. 3-month-old mice of the indicated genotypes were fasted for 5 hours. (C) Cold tolerance, measured as body temperature after housing at 4°C for 5 hours. *, p < 0.05; **, p < 0.01; ***, p < 0.001. (TIF) [file pgen.1006716.s003.tif]

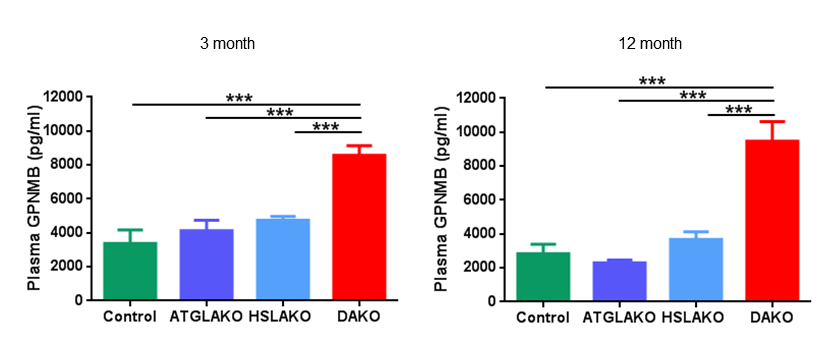

Supplement: S3 Fig — Gpnmb levels of 3- and 12-month-old mice of the indicated genotypes. *, p < 0.05; **, p < 0.01; ***, p < 0.001. (TIF) [file pgen.1006716.s004.tif]

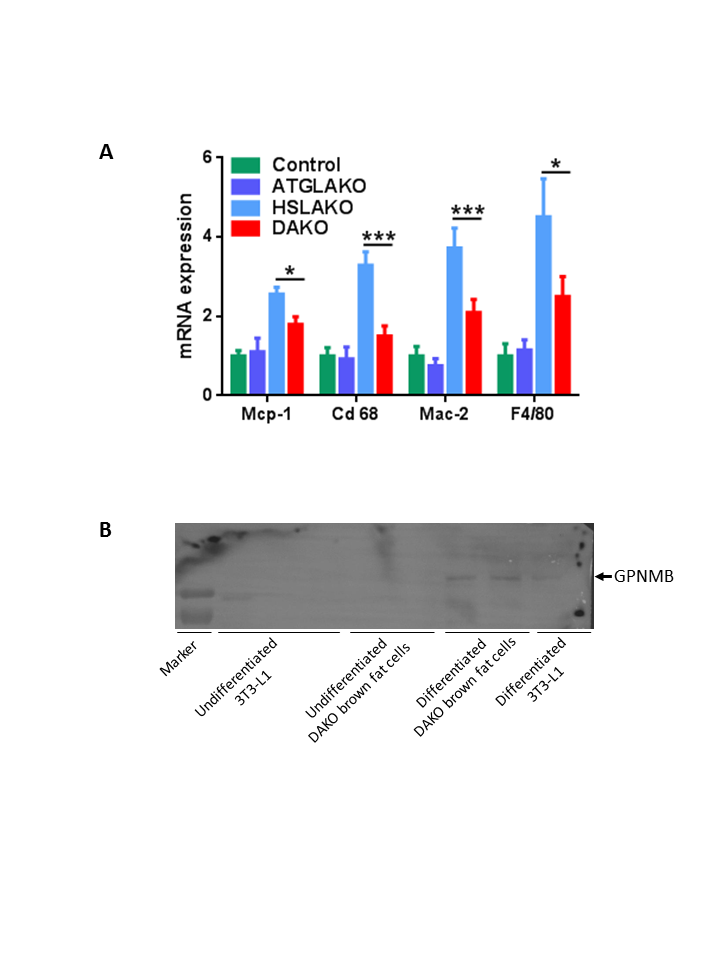

Supplement: S4 Fig — (A) Expression of macrophage marker mRNAs in brown adipose tissue of mice. (B) Western blot showing Gpnmb expression before and after in vitro differentiation, in cultured DAKO brown adipocytes from DAKO mice and in NIH-3T3 L1 cells. *, p < 0.05; **, p < 0.01; ***, p < 0.001. (TIF) [file pgen.1006716.s005.tif]

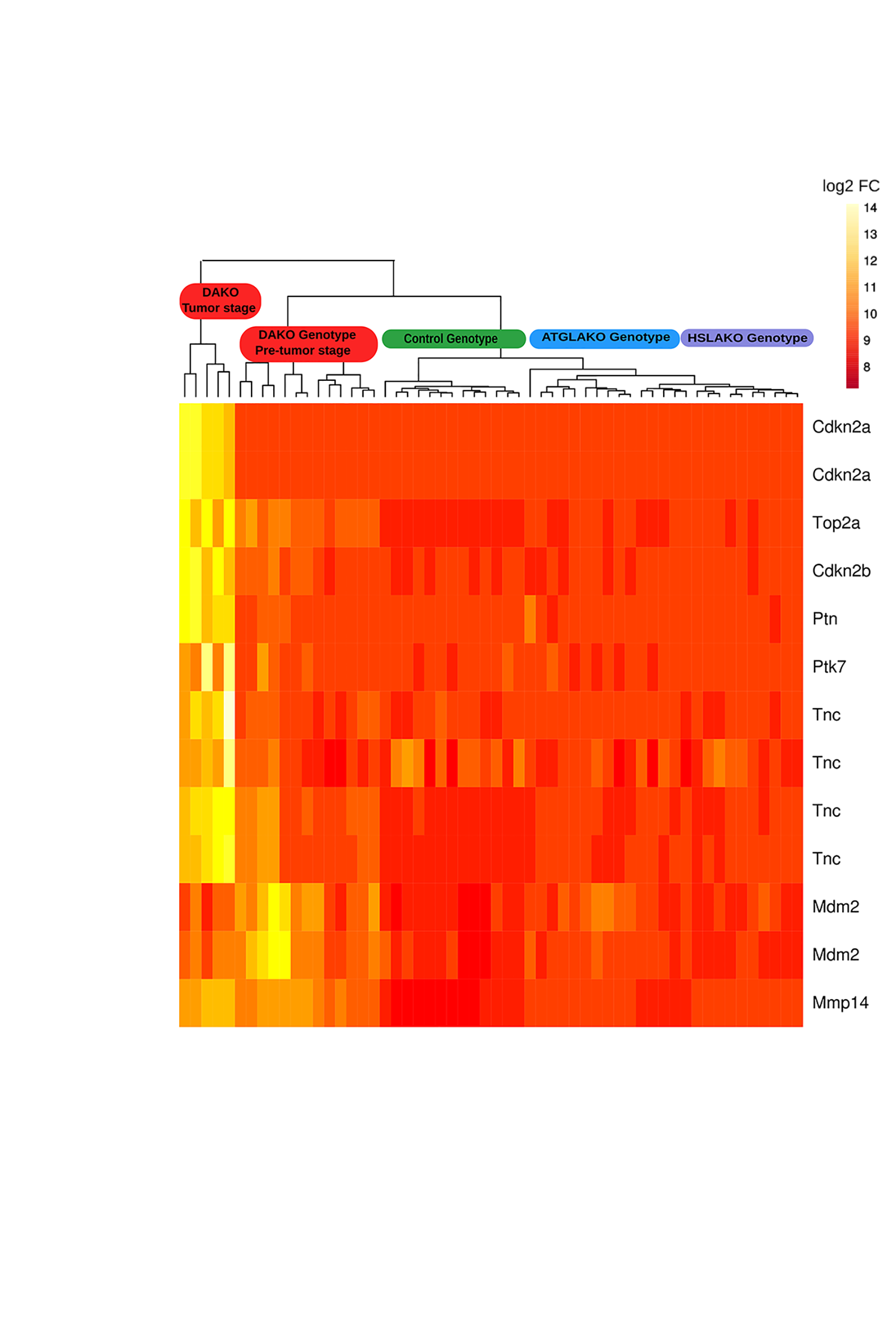

Supplement: S5 Fig — Heatmap showing the sarcomas and other cancer-associated gene expression in four genotypes of mice studied. Sample type (liposarcoma, BAT) and genotype are indicated in the dendrogram on the top. The genes in question are listed on the right. (TIF) [file pgen.1006716.s006.tif]

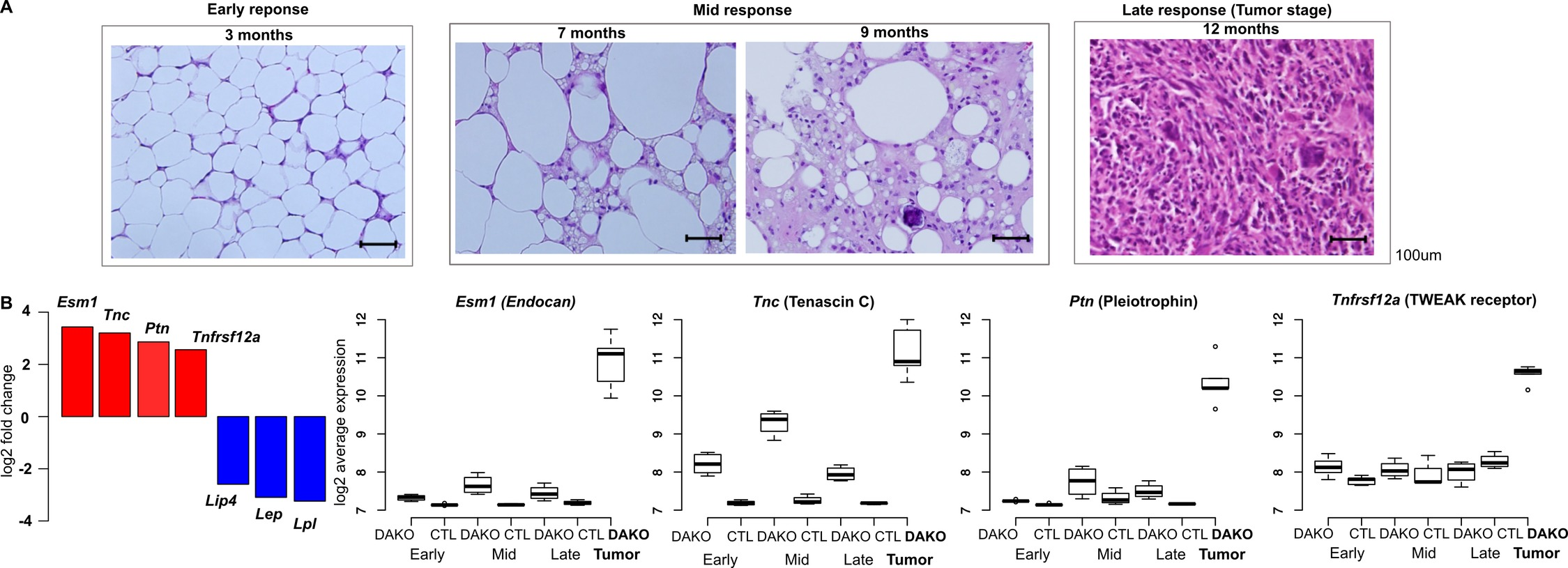

Supplement: S6 Fig — (A) Haematoxylin-eosin stained DAKO BAT at four time points (3, 7, 9 and 12 months). (B) Differential expression analysis, comparing pre-cancerous BAT and liposarcoma tissue, highlighting the most up- and down-regulated genes identified in transcriptome analysis. Candidate genes involved in angiogenesis are displayed as boxplots for four different time points: early (3 months), mid (7 months), late (12 months) and tumor (11–14 months). (TIF) [file pgen.1006716.s007.tif]

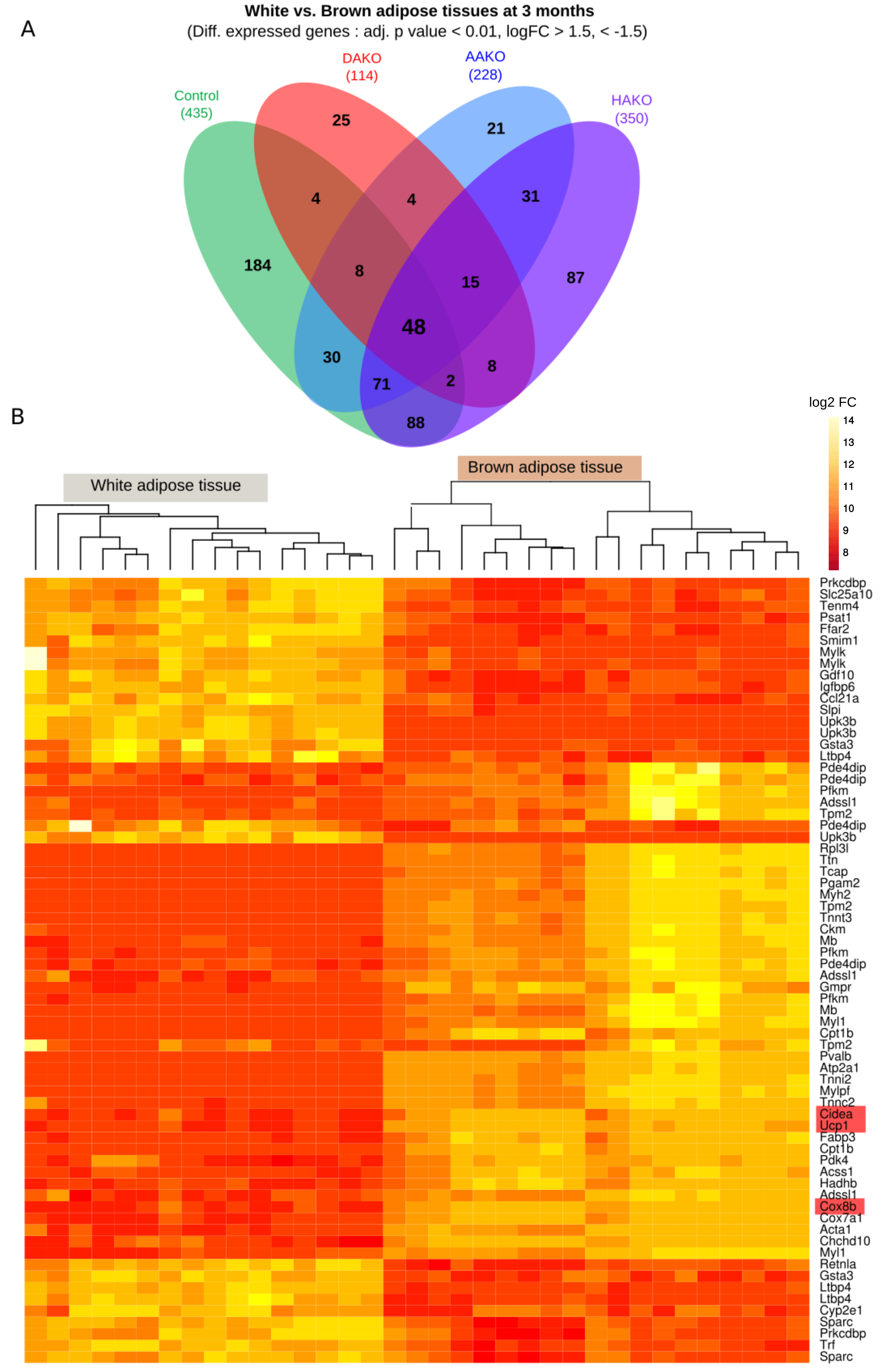

Supplement: S7 Fig — (A) Venn diagram highlighting differentially expressed genes between brown and white adipose tissues for all possible genotype combinations in three-month-old mice. (B) Heatmap displaying the subset of 48 differentially-expressed genes for all genotypes. Commonly-used markers for BAT (Ucp1, Cidea, Cox8b) are highlighted in red. (TIF) [file pgen.1006716.s008.tif]

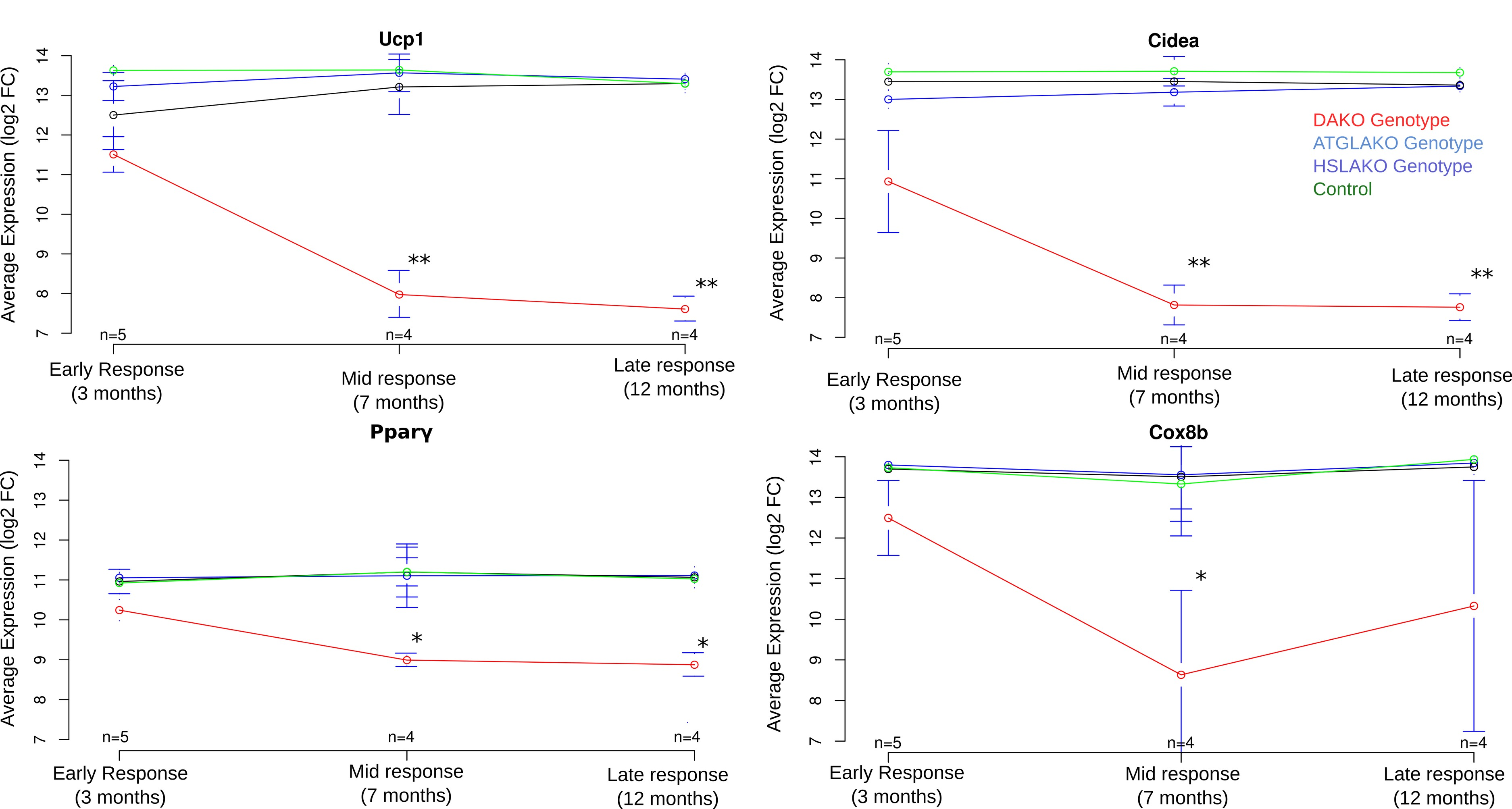

Supplement: S8 Fig — Time course analysis of BAT markers Ucp1, Cidea, Pparγ and Cox8b prior to tumor development in the course of 12 months. Significant down regulation of genes was assessed by comparing expression means using ANOVA. **, p < 0.01; *, p < 0.05. (TIF) [file pgen.1006716.s009.tif]

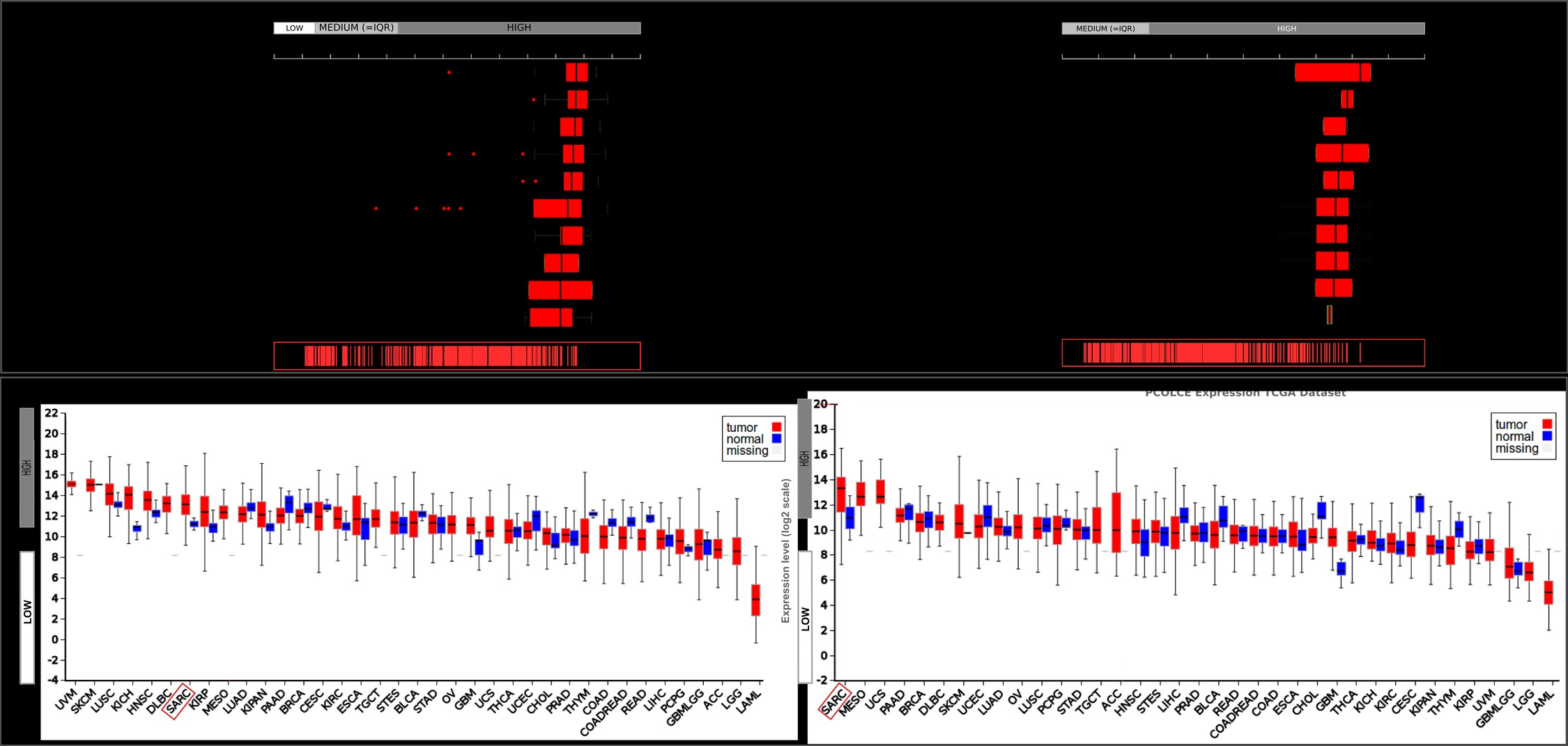

Supplement: S9 Fig — (A) Data from the genevisible dataset containing 497 different cancer expression profiles highlighting the 10 cancers with the highest expression of the two potential markers GPNMB and PCOLCE. (B) Data from the TCGA dataset, visualized by the Firebrowse website available through the Broad Institute indicating expression levels of GPNMB and PCOLCE across all available cancer subtypes. SARC, sarcoma (TIF) [file pgen.1006716.s010.tif]

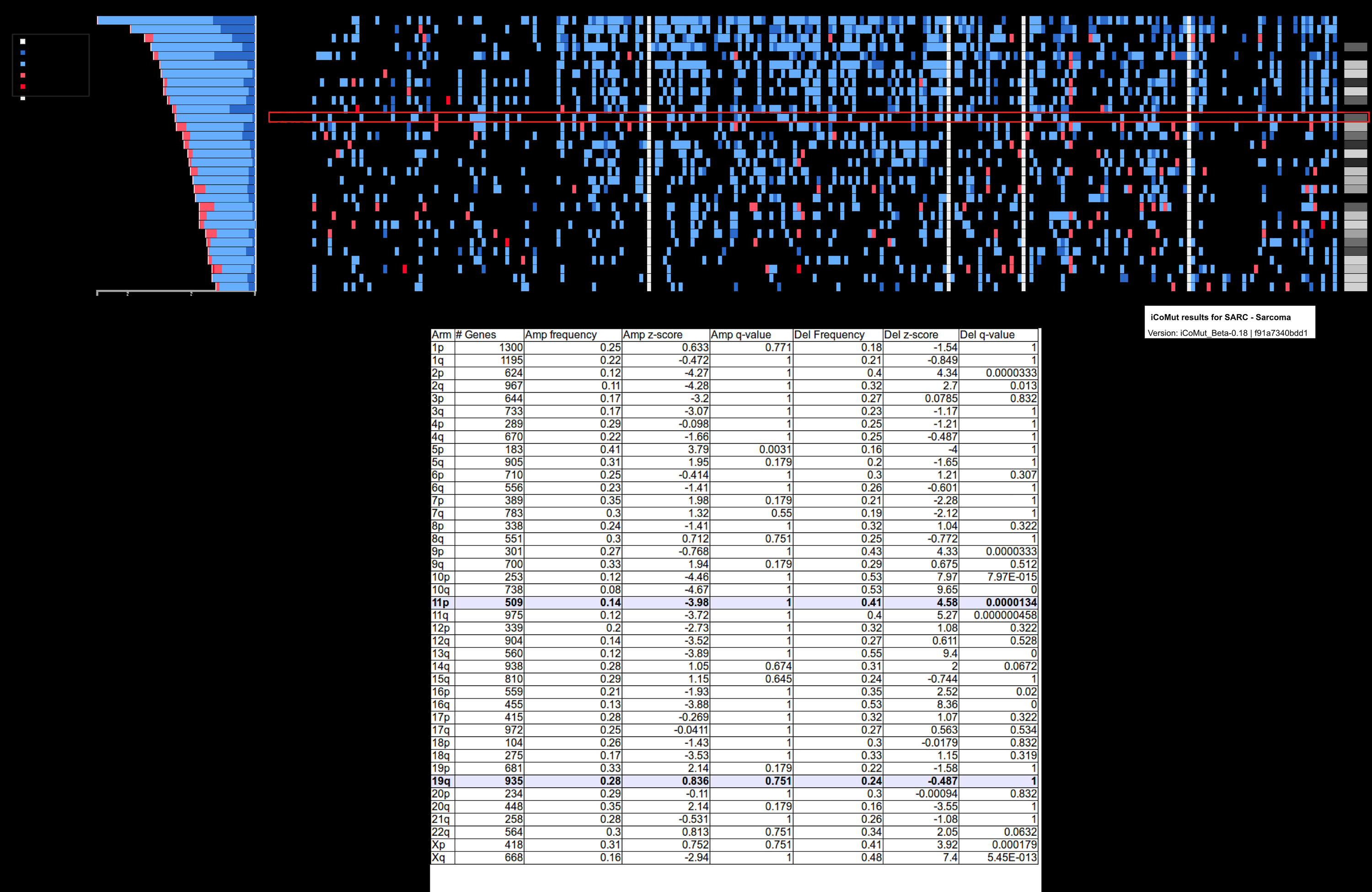

Supplement: S10 Fig — (A) Cosmic annotation of CNVs in 265 patients with soft tissue sarcomas. The 11p15.5 region containing PNPLA2 is boxed. (B) Chromosomal region 11p harboring PNPLA2 has a significantly increased deletion frequency. Chromosomal region 19q, containing LIPE, does not show copy number variation. (TIF) [file pgen.1006716.s011.tif]
